# The Novel TORC1/2 Kinase Inhibitor PQR620 Has Anti-Tumor Activity in Lymphomas as a Single Agent and in Combination with Venetoclax

**DOI:** 10.3390/cancers11060775

**Published:** 2019-06-04

**Authors:** Chiara Tarantelli, Eugenio Gaudio, Petra Hillmann, Filippo Spriano, Giulio Sartori, Luca Aresu, Luciano Cascione, Denise Rageot, Ivo Kwee, Florent Beaufils, Emanuele Zucca, Anastasios Stathis, Matthias P. Wymann, Vladimir Cmiljanovic, Doriano Fabbro, Francesco Bertoni

**Affiliations:** 1 Institute of Oncology Research, Università della Svizzera Italiana, 6500 Bellinzona, Switzerland; chiara.tarantelli@ior.usi.ch (C.T.); eugenio.gaudio@ior.usi.ch (E.G.); filippo.spriano@ior.usi.ch (F.S.); giulio.sartori@ior.usi.ch (G.S.); luciano.cascione@ior.usi.ch (L.C.); ivo.kwee@gmail.com (I.K.); 2 PIQUR Therapeutics AG, 4057 Basel, Switzerland; petra.hillmann@piqur.com (P.H.); fbeaufils@yahoo.fr (F.B.); vladimir.cmiljanovic@icloud.com (V.C.); doriano.fabbro@piqur.com (D.F.); 3 Dipartimento di Scienze Veterinarie, Università degli Studi di Torino, 10095 Grugliasco (TO), Italy; luca.aresu@unito.it; 4 Swiss Institute of Bioinformatics (SIB), 1015 Lausanne, Switzerland; 5 Department of Biomedicine, University of Basel, 4056 Basel, Switzerland; denise.rageot@unibas.ch (D.R.); matthias.wymann@unibas.ch (M.P.W.); 6 Oncology Institute of Southern Switzerland, 6500 Bellinzona, Switzerland; emanuelezucca@yahoo.com (E.Z.); anastasios.stathis@eoc.ch (A.S.)

**Keywords:** lymphoma, mTORC1, mTORC2, venetoclax, mantle cell lymphoma, diffuse large B cell lymphoma

## Abstract

The phosphatidylinositol 3-kinase (PI3K)/AKT/mammalian target of rapamycin (mTOR) signaling cascade is an important therapeutic target for lymphomas. Rapamycin-derivates as allosteric mTOR complex 1 (TORC1) inhibitors have shown moderate preclinical and clinical anti-lymphoma activity. Here, we assessed the anti-tumor activity of PQR620, a novel brain penetrant dual TORC1/2 inhibitor, in 56 lymphoma cell lines. We observed anti-tumor activity across 56 lymphoma models with a median IC_50_ value of 250 nM after 72 h of exposure. PQR620 was largely cytostatic, but the combination with the BCL2 inhibitor venetoclax led to cytotoxicity. Both the single agent and the combination data were validated in xenograft models. The data support further evaluation of PQR620 as a single agent or in combination with venetoclax.

## 1. Introduction

The high frequency of genomic alterations in lymphomas affecting genes coding for proteins involved in the phosphoinositide 3-kinase (PI3K)/AKT/mammalian target of rapamycin (mTOR) pathway [1,2] highlights the importance of this signaling cascade as a therapeutic target. mTOR is an atypical serine/threonine kinase that mediates a variety of signals that directly or indirectly regulate cellular growth and metabolism [3,4]. It is present in two complexes, mTOR complex 1 (TORC1) and mTOR complex 2 (TORC2), which differ for the presence of additional proteins. Acute exposure to rapamycin inhibits TORC1 but not TORC2. It is now recognized that not only TORC1 but also TORC2 is positively regulated by the PI3K signaling [3,4]. Importantly, the first generation of mTOR inhibitors, sirolimus (rapamycin), temsirolimus (CCI779), everolimus (RAD001) and ridaforolimus (AP23573/MK-8669), act as allosteric inhibitors blocking the interaction between mTOR and FBPK12 resulting in inhibition of TORC1 only [3,4]. These compounds are also known as rapalogs since they are chemically derived from rapamycin and maintain the same mechanism of action [3,4]. Rapalogs have shown preclinical and clinical anti-lymphoma activity [3,4,5,6]. In particular, temsirolimus demonstrated a higher response rate and progression free survival than investigators’ treatment choices in relapsed or refractory mantle cell lymphoma [7,8] and was approved by the European Medicines Agency (EMA) for patient populations [6]. However, the clinical activity of first generation mTOR inhibitors remains relatively limited and their use comes with side effects, as also demonstrated in two recently reported phase III clinical trials. The result of Temsirolimus was that it was less active and more toxic than the Bruton Tyrosine Kinase (BTK) inhibitor ibrutinib [9] and the addition of everolimus after R-CHOP (rituximab, cyclophosphamide, doxorubicin, vincristine and prednisone) did not improve the outcome of patients with newly diagnosed high-risk diffuse large B cell lymphoma (DLBCL) [10]. Biologically, the inability of rapalogs to inhibit TORC1 associated with the activation of pro-survival feedback loops are known limitations in the mechanism of action of these types of compounds [3,4]. One way to overcome these problems is the design of dual TORC1/2 inhibitors that target the catalytic site of mTOR [3,4,11,12,13], thus blocking the enzyme independently from its interacting proteins. Second generation mTOR inhibitors have shown stronger preclinical anti-tumor activity than allosteric mTOR inhibitors [3,4,11,12,13,14,15]. Results of the first reported phase I studies support this approach [3,4,16,17,18], although the toxicity profile can still represent an important issue [19,20]. Here, we report the anti-tumor activity of the brain penetrant dual TORC1/2 inhibitor PQR620 [13] as a single agent and in combination with venetoclax in lymphoma models.

## 2. Results

### 2.1. PQR620 Has In Vitro Anti-Lymphoma Activity

**Figure 1:**
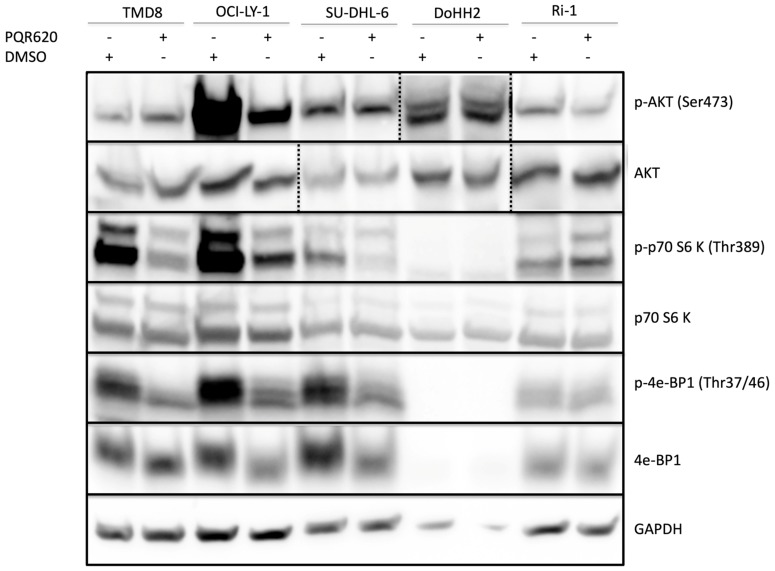
PQR620 affects TORC1/2 signaling pathways by downregulating p-AKT, p-p70 S6 and p-4e-BP1 in most cell lines. Two ABC-DLBCL (TMD8, Ri-1) and three GCB-DLBCL (OCI-LY-1, SU-DHL-6, DoHH2) cell lines were treated with PQR620 (2 µM, 24 h) or, as control, DMSO.

The anti-tumor activity of the novel TORC1/2 inhibitor PQR620 was assessed in a large panel of cell lines (*n* = 56) derived from lymphomas. The compound showed potent anti-proliferative activity in most of the tested cell lines (Table 1), with a median IC_50_ of 249.53 nM (95% C.I., 221–294). The most sensitive subtype was mantle cell lymphoma (MCL) (*p* = 0.0232 among all human cell lines; *p* = 0.0552 within B-cell lymphomas) (Table 2). Conversely, the ALK+ anaplastic large cell lymphomas (ALK+ALCL) were the least sensitive (*p* = 0.0095) (Table 2). When we focused on diffuse large B cell lymphomas (DLBCL), representing the largest group of cell lines, the presence of *MYC* or *BCL2* or the cell of origin did not affect the response to PQR620. However, DLBCL cell lines bearing *TP53* inactivation were less sensitive than *TP53* wild-type cells (300 nM (95% C.I., 242–364) vs. 136 nM (95% C.I., 74–233); *p* = 0.0007). The anti-tumor activity of PQR620 appeared mostly cytostatic. Apoptosis induction was only seen in 8/56 cell lines (14%: 95% C.I., 6–26%) without association with histotype, *BCL2*, *MYC* or *TP53* status (Table 1). PQR620 was able to act both on the TORC1 and TORC2 pathways. Immunoblotting of DLBCL cell lines exposed to PQR620 (2 µM, 24 h) showed reduction of p-p70 S6 (Thr389) and p-4e-BP1 (Thr37/46) levels, indicative of TORC1 inhibition, and of p-AKT (Ser 473), indicative of TORC2 inhibition (Figure 1).

### 2.2. PQR620 Has In Vivo Anti-Lymphoma Activity

**Figure 2:**
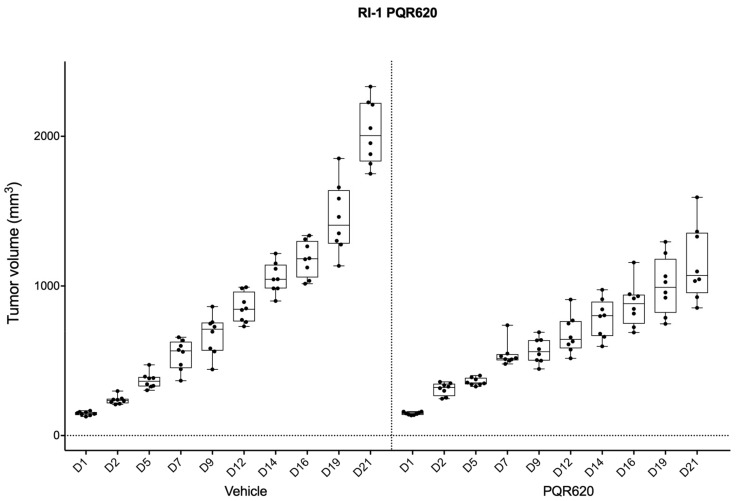
Effects of PQR620 as a single agent in a xenograft model of ABC-DLBCL. NOD-Scid mice subcutaneously inoculated with RI-1 (15 × 10^6^) cells were split into two groups respectively treated with PQR620 (50 mg/kg, 7 days/w, po, *n* = 8), and a control vehicle (*n* = 8). In each box-plot, the line in the middle of the box represents the median and the box extends from the 25th to the 75th percentile (interquartile range, IQ); the whiskers extend to the upper and lower adjacent values (i.e., ±1.5 IQ). PQR620 versus vehicle, D2, D12, D14, D16, D19, D21, *p* < 0.05.

The observed in vitro anti-tumor activity of PQR620 was then evaluated in an in vivo model using the activated B-cell-like (ABC) DLBCL RI-1 cell line. Treatments with PQR620 (100 mg/kg dose per day, one a day for 7 days/week (Qdx7/w)) started with 100–150 mm^3^ tumors and were carried out for 21 days. PQR620 determined a decrease of the tumor volumes in comparison with controls from day 12 (*p* < 0.05) (Figure 2). There was no toxicity observed (reported as body weight loss).

### 2.3. PQR620 Has In Vitro and In Vivo Synergism with the BCL2 Inhibitor Venetoclax

**Figure 3:**
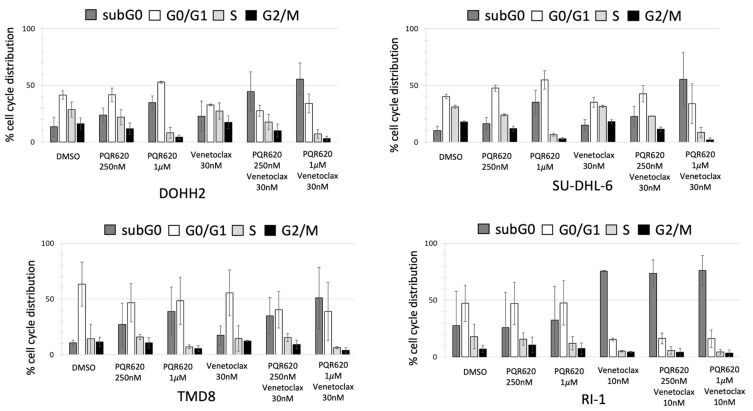
The combination of PQR620 and venetoclax are more in vitro cytotoxic than the single agents. Cell cycle distribution of four DLBCL cell lines (GCB: DOHH2, SU-DHL-6; ABC: TMD8, RI1) treated with two different concentrations of PQR620 (250 nM or 1 µM) and/or venetoclax.

Due to the low induction of apoptosis after PQR620 as a single agent, we assessed the combination of the dual TORC1/2 inhibitor and the BCL2 inhibitor venetoclax in four DLBCL cell lines. The combination was in vitro synergistic in terms of anti-proliferative effect, as shown by exposing the cell lines to increasing doses of PQR620 and venetoclax as single agents or in combination (Figure S1). The addition of venetoclax increased the cell death, as indicated by a higher percentage of cells in the subG0 phase, in all the cell lines but the RI-1 in which the BCL2-inhibitor as a single agent was already highly cytotoxic (Figure 3).

**Figure 4:**
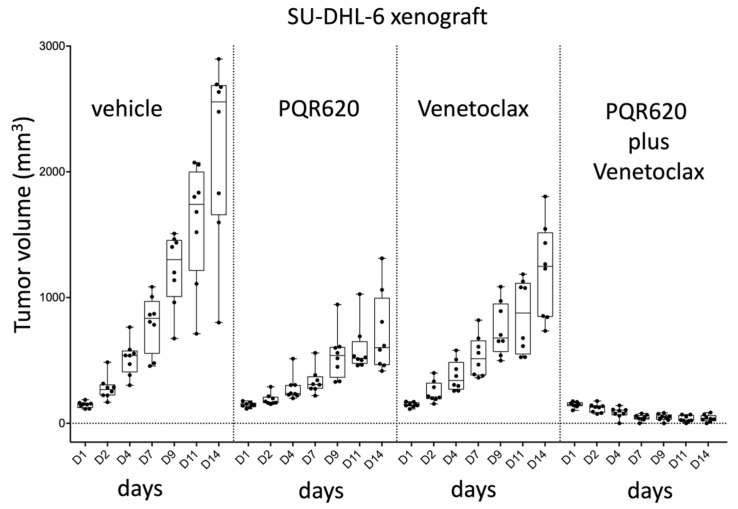
The combination of PQR620 and venetoclax have stronger in vivo anti-tumor activity than the single agents in a xenograft model of GCB-DLBCL. NOD-Scid mice subcutaneously inoculated with SU-DHL-6 (15 × 10^6^) cells were split into four groups respectively treated with PQR620 (100 mg/kg, Qdx7/w, po, *n* = 8), Venetoclax (100 mg/kg, Qdx7/w, a combination of PQR620 with venetoclax (*n* = 8) and control vehicle (*n* = 8). PQR620 versus vehicle, D4–D14, *p* < 0.01; PQR620+venetoclax versus vehicle, D2–D14, *p* < 0.01; PQR620+venetoclax versus PQR620, D2–D14, *p* < 0.01; PQR620+venetoclax versus venetoclax, D2–D14, *p* < 0.01. In each box-plot, the line in the middle of the box represents the median and the box extends from the 25th to the 75th percentile (interquartile range, IQ); the whiskers extend to the upper and lower adjacent values (i.e., ±1.5 IQ).

Based on the in vitro data, the activity of PQR620 (100 mg/kg dose per day, Qdx7/w, 21 days) in combination with venetoclax (100 mg/kg, Qdx7/w) was evaluated in an in vivo model using the germinal center B-cell type (GCB)-DLBCL cell line SU-DHL-6, bearing the t(14;18) chromosomal translocation (Figure 4). PQR620 determined a 2-fold decrease of the tumor volumes in comparison with controls. The combination of PQR620 with venetoclax showed highly significant differences either versus control or single agents during all days of the experiment (D4, D7, D9, D11, D14; *p* < 0.001), resulting in an eradication of the implanted tumors in the absence of toxicity. The treated/control ratio (T/C) was <10% and, according to the U.S. National Cancer Institute (NCI) rules, the drug combination is declared as very active [23].

## 3. Discussion

PQR620 is a novel dual TORC1/2 inhibitor with higher affinity for the enzymatic catalytic domain than previously reported second-generation compounds, such as INK128, CC223 and AZD2014 [13]. Here, we have shown that PQR620 had anti-tumor activity across 56 lymphoma models with a median IC_50_ value of 250 nM after 72 h of exposure, lower than what is reported in 66 cell lines derived from various solid tumors [13].

PQR620 was largely cytostatic, as is the case for other mTOR inhibitors in lymphomas [24,25,26]. However, when we combined the dual TORC1/TORC2 inhibitor with the BCL2 inhibitor venetoclax we observed an in vitro increase in cell death. Importantly, the benefit of the combination was validated in vivo upon treating xenografts derived from a GCB-DLBCL cell line with eradication of the tumor cells. Our data are in agreement with results obtained with venetoclax in combination with the other dual TORC1/TORC2, INK128, in acute myeloid leukemia [27], as well as data reported in lymphoma models with the BCL2 inhibitor combined with the dual PI3K/mTOR inhibitor bimiralisib (PQR309) [28] or different PI3K inhibitors [29,30,31,32]. The effect appears to be mediated by downregulation of anti-apoptotic proteins (MCL1 or BCLXL) mediated by the inhibition of the PI3K/AKT/mTOR pathway [29,31,32]. So far, in the clinical setting, the addition of venetoclax to other drugs seems feasible with no additional major toxicities [33,34,35].

For novel compounds, it is useful to identify groups of patients that might benefit the most from the treatment. Genomics studies have identified DLBCL subgroups with a constitutively activated PI3K/mTOR pathway: GCB- and ABC-DLBCL, belonging to the B-cell receptor signaling cluster [36], with GCB-DLBCL mostly falling into the newly described EZB subtype by Schmitz et al. [2] or Cluster 3 by Chapuy et al. [1]. Importantly, the available DLBCL cell lines recapitulate the genetic and biologic features of these clinical entities [32,36], and PQR620 was active in such models. These classifications should be incorporated in the next clinical trials exploring PQR620 or other members of the same class to identify the patients that benefit the most from the treatment.

The anti-tumor activity of PQR620 was particularly high in the MCL cell lines. This observation is interesting, since MCL is the histotype in which first generation mTOR inhibitors have been mostly clinically developed [3,4,8,37,38] with the European Medicines Agency (EMA) approval of temsirolimus for patients with relapsed/refractory disease [6,8]. Additionally, although they could not be defined as resistant, two groups of lymphomas presented reduced sensitivity to PQR620. ALK+ALCL presented a lower sensitivity than the other cell lines. In this tumor, the PI3K/AKT/mTOR pathway is downstream of ALK and constitutively active [39], but its inhibition alone might not be enough to efficiently target the lymphoma cells [40,41].

The second group with decreased PQR620 sensitivity was represented by mutated DLBCL cell lines bearing an inactive *TP53*. The contribution of TP53 in the response to mTOR inhibitors is not defined, but it is certainly relevant in particular conditions [24,42,43]. Data obtained in Eμ-Myc mouse lymphomas treated with everolimus clearly show that the lack of TP53 is a mechanism of primary resistance to the rapalog [24], indicating the importance of a TP53-mediated component in the mechanism of action of this class of compounds in lymphomas. Referring again to the newly described DLBCL subtypes, patients belonging to Cluster 2 [1], enriched in *TP53* mutations and deletions, do not represent the best target population for PQR620.

PQR620 was designed starting from the dual PI3K/mTOR inhibitor bimiralisib (PQR309) [44,45], increasing the molecule affinity for mTOR while decreasing binding to PI3K [13]. Bimiralisib has preclinical [28] and early clinical anti-lymphoma activity [46] in primary central nervous system lymphoma (PCNSL) [47]. PQR620 retains the ability of bimiralisib [44] to pass the blood-brain barrier [13]. This is relevant as PQR620 was active in ABC DLBCL cell lines, and the most common type, PCNSL, which is an aggressive type of extranodal lymphoma, for which PI3K/mTOR inhibitors are an area of clinical research [48]. Our data also show that PQ620 maintained the anti-tumor activity of bimiralisib reported in a canine DLBCL model [49], indicating that dogs with spontaneous lymphomas might represent an effective pre-clinical animal model to test this compound as well.

## 4. Materials and Methods

### 4.1. In Vitro Experiments

Established human cell lines were cultured as previously described [28] and their identity was authenticated by short tandem repeat (STR) DNA profiling (IDEXX BioResearch, Ludwigsburg, Germany). The status of *BCL2*, *MYC* and *TP53* was defined as previously reported [28]. PQR620 was provided by PIQUR Therapeutics and dissolved in dimethyl sulphoxide (DMSO) to obtain a stock concentration of 10 mM. Venetoclax was obtained from Selleckchem (Houston, TX, USA). Viability and caspase-3/7 activation were assessed with the ApoTox-Glo Assay (Promega, Madison, WI, USA), as previously reported [21]. Apoptosis was defined by at least a 1.5-fold increase in signal activation over controls [21] and it was calculated in our models at the concentration of 1500 nM of PQR620. The effect on cell proliferation, cell cycle analysis and protein extraction, separation and immunoblotting were done as previously described [28,50]. The following antibodies were used: AKT (CST 9272, Cell signaling Technology, Danvers, MA, USA), p-AKT (Ser 473) (CST 4060), p-p70 S6K (Thr 389) (CST 9205), p70 S6K (CST 9202), p-4e-BP1 (Thr 37/46) (CST 9459), 4e-BP1 (CST 9452) anti-GAPDH (Ebioscence FF26A). Associations in two-way tables were tested for statistical significance using either the *X*^2^ test or Fisher’s exact test (two-tailed), as appropriate. Binomial exact 95% confidence intervals (95% C.I.s) were calculated for median percentages. Differences in IC_50_ values among lymphoma subtypes were calculated using the Wilcoxon rank-sum test. Statistical significance was defined by *p* values of 0.05 or less. The synergism of drug combination was calculated by using the Chou–Talalay Index, as previously done [28]. Statistical analyses were performed using Stata/SE 12.1 for Mac (Stata Corporation, College Station, TX, USA), and boxplots with GraphPad Prism v. 7.0d (GraphPad Software, La Jolla, CA, USA).

### 4.2. In Vivo Experiments

Xenograft experiments with the RI-1 and the SU-DHL-6 cell lines were performed using NOD-Scid (NOD.CB17-*Prkdcscid*/NCrHsd) mice and analysed as previously described [28]. Mice maintenance and animal experiments were performed under the Guide of Animal Care and Use (NCR 2011) and under the Chinese National Standard (GB14925-2010) (Crown Bioscience Inc., Taicang City, China). These studies were part of two larger works assessing other agents; hence, the vehicle arm for the RI-1 xenograft and the vehicle and the venetoclax arms for the SU-DHL-6 xenograft have been previously reported [28]. In vivo toxicity was evaluated by weighing the mice 3 times per week. Tumor weight loss higher than 10% in one week was considered to be the main sign of toxicity due to the drug. Treated/control ratio (T/C) was calculated at the end of the study as follows: T/C = (median tumor volume in the treated group/median tumor volume in the vehicle control group) × 100. A × T/C ≤ 42% was declared active in agreement with NCI criteria [23].

## 5. Conclusions

In conclusion, PQR620 showed in vitro and in vivo anti-tumor activity in lymphoma models as a single agent and in combination with venetoclax, supporting its further development.

## Figures and Tables

**Table 1:** Anti-tumor activity of PQR620 in lymphoma cell lines. The IC_50_ was calculated after 72 h of drug exposure. Apoptosis was defined by at least a 1.5-fold increase in signal activation with respect to controls. *BCL2*, *MYC* and *TP53* status were defined as previously reported [21].

Cell Line	Histology	IC50 (nM)	Apoptosis Induction (At 1500 nM)	BCL2 Translocation	MYC Translocation	TP53 Inactive
**CLBL-1**	Canine DLBCL	267.38	no	n.a.	n.a.	n.a.
**DB**	GCB-DLBCL	507.79	no	1	0	
**DOHH2**	GCB-DLBCL	144.03	no	1	1	0
**ESKO-L**	MZL	378.09	no	n.a.	0	n.a.
**FARAGE**	GCB-DLBCL	246.32	no	0	0	1
**FE-PD**	PTCL-NOS	261.14	no	n.a.	n.a.	n.a.
**GRANTA519**	MCL	296.25	no	0	0	1
**H9**	CTCL	614.19	no	n.a.	n.a.	n.a.
**HAIR-M**	MZL	332.6	no	n.a.	0	n.a.
**HBL1**	ABC-DLBCL	370.8	no	n.a.	0	n.a.
**HC1**	MZL	170.76	no	n.a.	0	n.a.
**HH**	CTCL	128.83	no	n.a.	n.a.	n.a.
**HUT-78**	CTCL	1396.59	no	n.a.	n.a.	n.a.
**JEKO1**	MCL	234.54	no	0	0	1
**JVM2**	MCL	131.66	no	0	0	0
**KARPAS1106-P**	PMBCL	322.89	yes	n.a.	0	n.a.
**KARPAS1718**	MZL	735.31	no	n.a.	0	1
**KARPAS299**	ALCL, ALK+	425.69	no	n.a.	n.a.	1
**KARPAS422**	GCB-DLBCL	150.77	no	1	0	1
**KI-JK**	ALCL, ALK+	456.52	no	n.a.	n.a.	n.a.
**L82**	ALCL, ALK+	364.12	no	n.a.	n.a.	1
**MAC1**	ALCL, ALK−	233.13	no	n.a.	n.a.	n.a.
**MAVER1**	MCL	91.78	no	n.a.	1	1
**MEC1**	CLL	348.52	no	n.a.	0	1
**MINO**	MCL	120.78	no	n.a.	1	1
**OCI-LY-1**	GCB-DLBCL	314.96	no	1	0	1
**OCI-LY-10**	ABC-DLBCL	220.15	no	0	0	1
**OCI-LY-18**	GCB-DLBCL	285.09	no	1	1	1
**OCI-LY-19**	GCB-DLBCL	128.81	no	1	n.a.	0
**OCI-LY-3**	ABC-DLBCL	127.2	no	0	0	0
**OCI-LY-7**	GCB-DLBCL	107.01	no	0	1	1
**OCI-LY-8**	GCB-DLBCL	210.4	no	1	1	1
**PCL12**	CLL	109.21	no	n.a.	0	n.a.
**PFEIFFER**	GCB-DLBCL	1069.2	no	1	0	1
**RCK8**	GCB-DLBCL	5.05	yes	n.a.	0	0
**REC1**	MCL	80.53	no	n.a.	0	1
**RI-1**	ABC-DLBCL	346.24	no	n.a.	1	1
**SP49**	MCL	250	yes	n.a.	0	n.a.
**SP53**	MCL	149.61	no	n.a.	0	n.a.
**SSK41**	MZL	200.25	no	n.a.	0	n.a.
**SU-DHL-1**	ALCL, ALK+	670.6	yes	n.a.	n.a.	1
**SU-DHL-10**	GCB-DLBCL	186.81	no	1	1	1
**SU-DHL-16**	GCB-DLBCL	249.06	no	n.a.	n.a.	n.a.
**SU-DHL-2**	ABC-DLBCL	320.52	no	0	0	n.a.
**SU-DHL-4**	GCB-DLBCL	226.36	no	1	0	1
**SU-DHL-5**	GCB-DLBCL	166.23	yes	0	0	n.a.
**SU-DHL-6**	GCB-DLBCL	398.97	no	1	0	1
**SU-DHL-8**	GCB-DLBCL	396.7	no	0	1	n.a.
**TMD8**	ABC-DLBCL	161.29	yes	n.a.	0	0
**TOLEDO**	GCB-DLBCL	349.22	yes	1	1	1
**U2932**	ABC-DLBCL	239.77	yes	0	0	1
**UPN1**	MCL	253.94	no	n.a.	0	1
**VAL**	GCB-DLBCL	261.31	no	1	1	0
**VL51**	MZL	215.97	no	n.a.	0	n.a.
**WSU-DLCL2**	GCB-DLBCL	264.18	no	1	0	1
**Z138**	MCL	237.47	no	n.a.	1	n.a.

DLBCL, diffuse large B-cell lymphoma; ABC-DLBCL, activated B-cell like diffuse large B-cell lymphoma; GCB-DLBCL, germinal center B-cell type diffuse large B-cell lymphoma; MCL, mantle cell lymphoma; ALCL, ALK+, ALK positive anaplastic large cell lymphoma; MZL, marginal zone lymphoma; CTCL, cutaneous T cell lymphoma; CLL, chronic B-cell leukemia; cALCL, cutaneous anaplastic large cell lymphoma; PMBCL, primary mediastinal large B-cell lymphoma; PTCL-NOS, peripheral T cell lymphoma, not otherwise specified; n.a. not available.

**Table 2:** Anti-tumor activity of PQR620 based on histology.

Histology	Median IC_50_ (nM)	95% Conf. Interval	Number of Cell Lines
ABC-DLBCL	230	88–354	8
GCB-DLBCL	249	181–325	19
Canine DLBCL	267	n.d.	1
MCL	192	101–253	10
MZL	274	174–700	6
PMBCL	323	n.d.	1
CLL	229	109–349 *	2
ALCL, ALK+	441	364–671 *	4
CTCL	614	129–1370 *	3
PTCL-NOS	261	n.d.	1
cALCL, ALKneg	233	n.d.	1

DLBCL, diffuse large B-cell lymphoma; ABC-DLBCL, activated B-cell like diffuse large B-cell lymphoma; GCB-DLBCL, germinal center B-cell type diffuse large B-cell lymphoma; MCL, mantle cell lymphoma; ALCL, ALK+, ALK positive anaplastic large cell lymphoma; MZL, marginal zone lymphoma; CTCL, cutaneous T cell lymphoma; CLL, chronic B-cell leukemia; cALCL, cutaneous anaplastic large cell lymphoma; PMBCL, primary mediastinal large B-cell lymphoma; PTCL-NOS, peripheral T cell lymphoma, not otherwise specified. * Lower confidence limit held at minimum (maximum) of sample [22]. n.d., not determined.

## Supplementary Materials

The following are available online at https://www.mdpi.com/2072-6694/11/6/775/s1. Figure S1: PQR620 was additive/synergistic when combined with venetoclax. Distribution of Chou-Talalay Combination Index (C.I.) values obtained in four DLBCL cell lines treated with different concentrations of PQR620 in combination with venetoclax. Y-axis, C.I. (C.I. < 0.9, synergism; 0.9 < C.I. < 1.1, additive effect).
