# Supplementary material for: The Novel TORC1/2 Kinase Inhibitor PQR620 Has Anti-Tumor Activity in Lymphomas as a Single Agent and in Combination with Venetoclax
Source: Cancers (Basel). 2019 Jun 4;11(6):775. doi: 10.3390/cancers11060775 (PMC6627883; doi:10.3390/cancers11060775)
Supplement: Supplementary file 1 [file cancers-11-00775-s001.pdf]

cancers

Supplementary Materials: The Novel TORC1/2 Kinase Inhibitor PQR620 Has Anti-Tumor Activity in Lymphomas as a Single Agent and in Combination with Venetoclax

Chiara Tarantelli, Eugenio Gaudio, Petra Hillmann, Filippo Spriano, Giulio Sartori, Luca Aresu, Luciano Cascione, Denise Rageot, Ivo Kwee, Florent Beaufils, Emanuele Zucca, Anastasios Stathis, Matthias P. Wymann, Vladimir Cmiljanovic, Doriano Fabbro and Francesco Bertoni

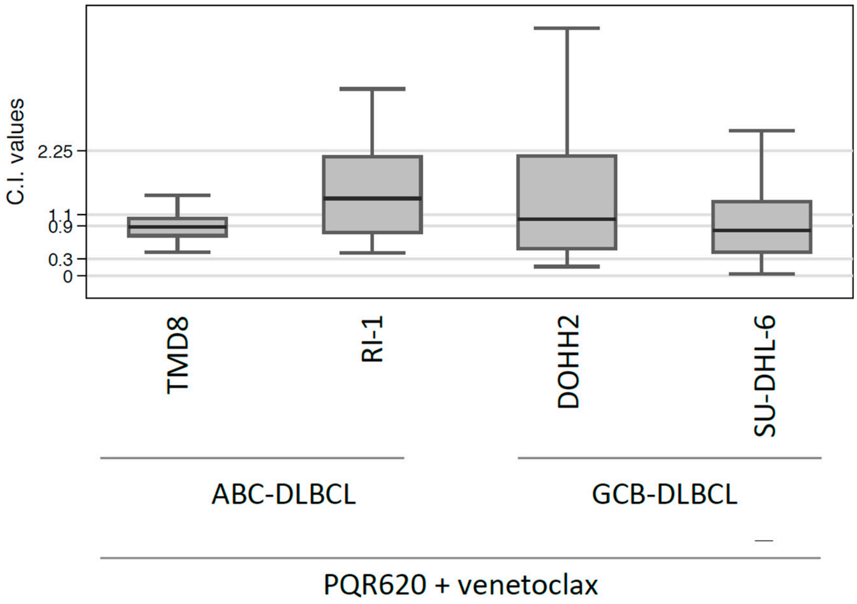

Figure S1. PQR620 was additive/synergistic when combined with venetoclax. Distribution of Chou-Talalay Combination Index (C.I.) values obtained in four DLBCL cell lines treated with different concentrations of PQR620 in combination with venetoclax. Y-axis, C.I. (C.I. < 0.9, synergism ; 0.9 < C.I. < 1.1, additive effect).

© 2019 by the authors. Licensee MDPI, Basel, Switzerland. This article is an open access article distributed under the terms and conditions of the Creative Commons Attribution (CC BY) license (http://creativecommons.org/licenses/by/4.0/).
